# Ventilator management in the age of COVID-19: response to “Logistic and organizational aspects of a dedicated intensive care unit for COVID-19 patients”

**DOI:** 10.1186/s13054-020-03069-0

**Published:** 2020-06-11

**Authors:** Alastair E. Moody, Bryce D. Beutler, Daniel Antwi-Amoabeng, Eric X. Lu, Charles E. Willyard, Irtqa Ilyas, Nageshwara Gullapalli

**Affiliations:** 1 Department of Anesthesiology, University of Utah, Salt Lake City, USA; 2 Department of Internal Medicine, Reno School of Medicine, University of Nevada, 1155 Mill Street, W-11, Reno, NV 89052 USA

Letter to the Editor in response to “Logistic and organizational aspects of a dedicated intensive care unit for COVID-19 patients” [1]:

In the age of coronavirus disease-19 (COVID-19), conservation of personal protective equipment (PPE) represents an urgent public health priority. Vargas et al. describe a logistic project and organizational plan to prevent the in-hospital spread of COVID-19 [1]. We build upon their approach by discussing another strategy to reduce infection among healthcare workers in the intensive care unit.

Ventilator settings are manipulated at bedside at least two to four times daily [2]; this requires close patient contact for several minutes on each occasion. Data on coronaviridae suggest that the risk of transmission is directly proportional to the duration of exposure in the absence of PPE [3]. Decreasing direct patient contact may therefore reduce hospital-acquired infections. Furthermore, bedside management of ventilator settings requires PPE that must be discarded after each use; this can rapidly deplete the number of available respirators and other items in short supply.

We propose an inexpensive and scalable mechanism of remote ventilator management that would allow healthcare providers to manipulate settings from an area outside of the patient room. This can be achieved using standard equipment using a simple modification: a commercially available extension cable can be used to relocate the ventilator display monitor to a sterile room (Fig. 1). Advantages of this strategy are outlined below:

1. Remote monitoring and management of ventilator settings can be performed without the use of PPE, thereby conserving respirators, gloves, and gowns for essential tasks that must be performed at bedside.
2. PPE donning and doffing is associated with high contamination rates [4]. Limiting the number of times PPE is donned and doffed reduces the risk of disease transmission. Furthermore, eliminating the need for PPE saves time, allowing providers to tend to other duties.
3. Multiple providers—including physicians, nurses, and respiratory therapists—can safely and easily monitor ventilator settings at all times with minimal exposure to pathogens.

**Fig. 1:**
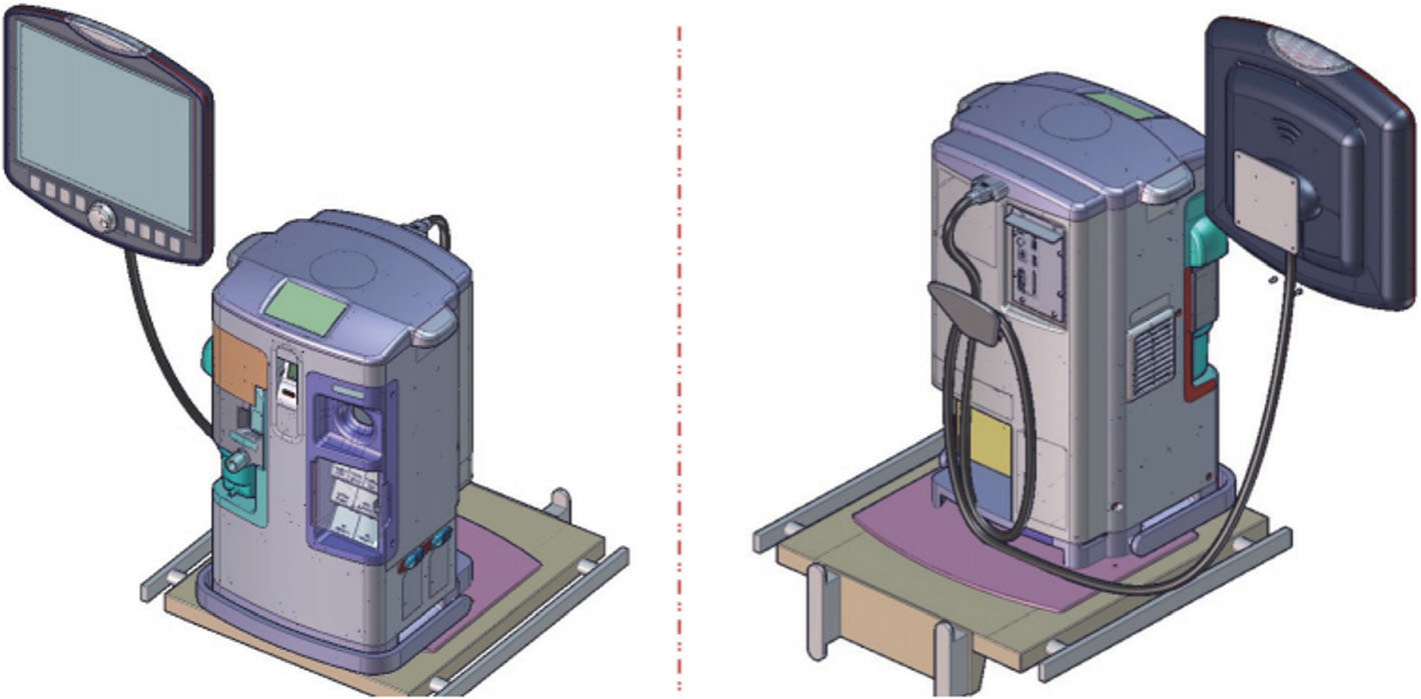
A commercially available cable can be used to relocate the ventilator display monitor to a sterile room. Artist rendition courtesy of *Medtronic Covidien 980* user manual and is the intellectual property of *Medtronic*

The concept outlined above can be extended to other critical components of patient care. For example, moving intravenous towers to a sterile room allows for management without the use of PPE. These interventions can limit exposure to essential bedside examinations.

Supportive care revolves largely around managing ventilator settings and monitoring vital signs, both of which can conceivably be performed remotely from a nearby sterile room. Our remote monitoring strategy has the potential to conserve a significant quantity of PPE throughout the course of this pandemic while ensuring appropriate patient care.

## Data Availability

N/A
